# The Impact of Drugs on Hydrogen Sulfide Homeostasis in Mammals

**DOI:** 10.3390/antiox12040908

**Published:** 2023-04-11

**Authors:** Asrar Alsaeedi, Simon Welham, Peter Rose, Yi-Zhun Zhu

**Affiliations:** 1 School of Biosciences, University of Nottingham, Loughborough, Leicestershire LE12 5RD, UK; stxaa105@nottingham.ac.uk (A.A.); simon.welham@nottingham.ac.uk (S.W.); 2 State Key Laboratory of Quality Research in Chinese Medicine, School of Pharmacy, Macau University of Science and Technology, Macau, China

**Keywords:** hydrogen sulfide, pharmacological drugs, health

## Abstract

Mammalian cells and tissues have the capacity to generate hydrogen sulfide gas (H_2_S) via catabolic routes involving cysteine metabolism. H_2_S acts on cell signaling cascades that are necessary in many biochemical and physiological roles important in the heart, brain, liver, kidney, urogenital tract, and cardiovascular and immune systems of mammals. Diminished levels of this molecule are observed in several pathophysiological conditions including heart disease, diabetes, obesity, and immune function. Interestingly, in the last two decades, it has become apparent that some commonly prescribed pharmacological drugs can impact the expression and activities of enzymes responsible for hydrogen sulfide production in cells and tissues. Therefore, the current review provides an overview of the studies that catalogue key drugs and their impact on hydrogen sulfide production in mammals.

## 1. Introduction

**Figure 1:**
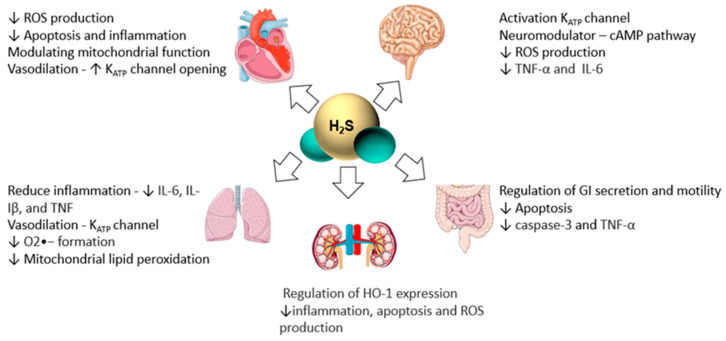
The physiological roles of H_2_S in mammalian systems. In cells and tissues, H_2_S is synthesized primarily from cysteine via cystathionine γ-lyase (CSE), cystathionine β synthetase (CBS), or 3-mercaptopyruvate sulfurtransferase (3-MST) in the transsulfuration pathway. Once produced, this gas can activate a spectrum of biochemical and physiological processes in cells to mediate effects in the brain, cardiovascular system, and other sites important for proper homeostatic function in vivo.

Understanding the physiological roles of gaseous mediators such as hydrogen sulfide (H_2_S) has been the focus of the past two decades of active research (for reviews, see [1,2,3,4]). It is now known that animals, plants, fungi, and bacteria naturally produce H_2_S and use this molecule in a range of biochemical and physiological processes [5]. In mammalian systems, H_2_S acts in cells and tissues and plays roles in mitochondrial function and homeostasis [6], cryoprotection [7], cellular antioxidant function [8], inflammation and inflammatory signaling [9], and tissue repair and wound healing [10]. Other research has shown important functions in the cardiovascular system [11,12]; the brain [13], kidney [14], and bladder [15]; the digestive [16] and urogenital tracts [17]; the respiratory and reproductive systems, such as erectile function [18,19,20]; obesity [21]; longevity and aging-related diseases [22]; fundamental cellular processes such as the cell cycle and apoptosis [23,24]; and the induction of signaling cascades such as p53, NF-κB, and Nrf2 [25,26,27] (summarized in Figure 1).

H_2_S is a signaling molecule in mammals, and it is therefore important to understand the biotic and abiotic stimuli that can lead to changes in the levels of this molecule in cells and tissues. Interestingly, several commonly prescribed pharmacological drugs have been reported to alter hydrogen sulfide levels in various model systems [28,29], and evidence relating to these molecules is described herein.

### 1.1. Hydrogen Sulfide (H_2_S) in Humans

#### 1.1.1. H_2_S Biosynthesis and Catabolism

Mammals generate H_2_S largely through two main enzymatic processes: cystathionine beta synthetase (CBS, EC 4.2.1.22) and cystathionine γ-lyase (CSE, EC 4.4.1.1) [1,30]. CBS protein is expressed in the brain, liver, kidney, and nervous system [31], and the CSE protein is expressed in the cardiovascular system and liver, respectively [32]. In addition, H_2_S can also be produced in a coupled reaction between 3-mercaptopyruvate sulfurtransferase (3-MST, EC 2.8.1.2) and the enzyme cysteine aminotransferase enzyme (CAT, EC 2.6.1.3), enzymes located in the mitochondria [33,34,35]. Once produced, H_2_S orchestrates its effects via the activation of cell signaling pathways, the activation of ion channels, or by promoting sulfhydration of protein targets. Alternatively, H_2_S is degraded via oxidative processes to thiosulfate (S_2_O_3_^2−^), then sulphate (SO_4_^2−^). The major enzymes mediating the detoxification of H_2_S are ethylmalonic encephalopathy protein 1 (ETHE1, EC 1.13.11.18), mitochondrial sulfide–quinone oxidoreductase (SQR, EC 1.8.5.4), thiosulfate sulfurtransferase (TST, EC 2.8.1.1), and sulfite oxidase (SO, EC 1.8.3.1). First, SQR cysteine persulfide is formed. Sulfane can further migrate to glutathione to form glutathione persulfide, or to sulfites and then to thiosulfates. Glutathione persulfide is oxidized by ETHE1, thiosulfate is oxidized by TST to regenerate sulfites, and sulfites are then oxidized to sulphates by SO [36,37]. A portion of H_2_S is also exhaled or scavenged by methemoglobin to generate sulfhemoglobin in the blood [38,39].

In addition to classical enzymatic routes of synthesis, H_2_S can also be generated via thiosulfate and polysulfide species [40,41]. These chemical routes are complex and mediated via cellular thiol exchange reactions with the sulfur amino acids and the redox molecule glutathione. These chemical routes are partly responsible for the health-promoting properties of some allium and brassica species [42].

#### 1.1.2. H_2_S Signaling

H_2_S is the third gaseous mediator found in mammals, akin to nitric oxide (NO) and carbon monoxide (CO) [11]. H_2_S can act as a scavenger of free radicals [8], direct post-transcriptional modification of cellular proteins via S-sulfhydration [43,44], activate ATP-sensitive potassium channels (KATP) [45] and transient receptor potential (TRP) channels [15], or function as a signaling molecule in numerous pathways.

Indeed, H_2_S acts on various kinases (reviewed in [1]), such as the p38 mitogen-activated protein kinase (p38 MAPK) [46], extracellular signal-regulated kinase (ERK) [47], Akt [48], protein kinase C (PKC) [49], c-Jun NH_2_-terminal kinase (JNK) [50], nuclear factor erythroid 2-related factor 2 (Nrf-2) [51], AMP-activated protein kinase [52], NAD-dependent deacetylase sirtuin-1 (SIRT1) [53], SIRT3 [25], and mechanistic target of rapamycin (mTOR) [54].

#### 1.1.3. Diminished Levels of H_2_S Occurs in Diseases

In humans, disease conditions may be exacerbated by changes in endogenous H_2_S production via one of several enzymatic pathways [55]. Most of these diseases have been associated with the loss in capacity of cells and tissues to generate H_2_S; this can result in decreased activity or expression of CSE, CBS, or a combination of the two [56]. Currently, diminished levels of H_2_S have been reported to occur in hypertension [57,58], vascular inflammation in pulmonary hypertension [59], tissue inflammation [60], atherosclerosis [61], diabetes [62,63], coronary heart diseases [64], gastric mucosal injury [65], sexual dysfunction [66], neurodegenerative conditions such as Alzheimer disease [67], dementia [68], and Parkinson’s disease [69]. H_2_S levels are also reduced in chronic kidney diseases [70], pulmonary tissues in children [71], and reperfusion injury [72]. Interestingly, replenishment with H_2_S via the use of sodium hydrosulfide (NaHS) or H_2_S donor molecules such as GYY4137 often improves the severity of these conditions in models of disease [73,74,75,76].

### 1.2. The Impacts of Pharmacological Drugs on Hydrogen Sulfide Homeostasis

Changes in the homeostatic levels of H_2_S occur naturally in the cells and tissues of mammals and are heavily influenced by disease status (reviewed in [2]), genetics [55,58], age [77,78], diet [55], and stress [22]. Less widely reported is the influence of conventional pharmacological drugs on H_2_S-generating systems in mammals. Several widely known medications have been reported to impact on the expression and activities of several components of the H_2_S-generating system in mammals [79,80,81]. The current review describes these therapeutic molecules and their effects on H_2_S biosynthetic routes in mammalian systems. See Table 1 for additional details of the studies included in this review [Table S1].

#### 1.2.1. Nonsteroidal Anti-Inflammatory Drugs (NSAIDs)

Worldwide, an estimated 30 million people take NSAIDs per day [105]. Non-steroid anti-inflammatory drugs (NSAIDs) are commonly used to relieve pain and reduce inflammation [106]. These properties are largely due to the impacts of these drugs on the gene transcription and protein synthesis of several inflammatory proteins such as cyclooxygenases (COX, EC 1.14.99.1) [107,108]. Unfortunately, common side effects when these drugs are administered chronically and used long term include gastrointestinal (GI) damage, as well as impacts on the cardiovascular system and kidney [109]. Recently, several researchers have reported that a number of NSAIDs reduce H_2_S production in mammalian systems. The suppression of H_2_S appears to be associated with reduced tissue healing and increased inflammation [110,111]. This realization has led several researchers to develop H_2_S-releasing NSAID hybrid compounds, of which many have reduced side effects by virtue of their capacity to sustain H_2_S levels at physiologically relevant concentrations. The aspects of this research are addressed below.

A plethora of animal models have examined the effects of NSAIDs on H_2_S levels in biological fluids and the impacts on the expression of biosynthetic enzymes in tissues. Recently, it was shown that Ketoprofen administered intragastrically to rats for 7 days caused significant reductions in H_2_S levels in the gastric and intestinal mucosa, leading to GI toxicity. In addition, Ketoprofen treatment altered the intestinal microbiome profile of animals, as well as the expression of the mammalian target of rapamycin (mTOR) and suppressor of the cytokine signaling 3 (SOCS3) pathways. In comparison, in the same model, the H_2_S-releasing donor compound ATB-352) significantly reduced GI tract damage via the preservation of H_2_S levels and suppression of oxidative/inflammatory response pathways in treated animals. It was noted that ATB-352 had an improved safety profile and preserved GI mucosal integrity by inhibiting inflammation in tissues [91].

Several other H_2_S-releasing NSAIDs have now been developed and compared to conventional NSAIDs. Again, many of these novel H_2_S-releasing NSAIDs have clear improvements in safety and efficacy when compared to their parental compounds. Indeed, the H_2_S-releasing analgesic/anti-inflammatory drug (ATB-346) has successfully passed phase two clinical double-blinded trials and is reported to have reduced GI toxicity [112]. Importantly, ATB-346 increased plasma H_2_S levels, which may have contributed to the cytoprotective effects. The impacts of other NSAIDs on H_2_S biosynthetic systems have also come under the spotlight over the last two decades. Magierowski et al. [89] reported that in Wistar rats pre-treated with Naproxen, a significant increased gastric mucosal protein expression of CSE was observed when compared to controls, while no effect was noticed on CBS and 3-MST [89]. The elevations in CSE were proposed to be a defensive mechanism of gastric tissues to the gastric damaging effect induced by Naproxen treatment. Naproxen significantly reduced gastric blood flow compared to controls, while pre-treatment with an H_2_S-releasing Naproxen derivative (ATB-346), or naproxen combined with NaHS, were seen to significantly increase gastric blood flow in rats when compared to Naproxen-treated rats. ATB-346 lowered plasma concentrations of IL-6 and TNF-α as compared with Naproxen-treated group. ATB-346 had a protective effect, which could be due to H_2_S formation, leading to activation of the Nrf-2/HO-1 pathway.

Other research has indicated that the gastrointestinal expression of CSE is reduced in mice treated with Aspirin (ASP), Indomethacin, Diclofenac, or Ketoprofen [81]. These researchers have indicated that reduced CSE expression in the GI tract enhances the susceptibility of FXR^−/−^ mice to damages caused by ASP and NSAIDs. FXR is a bile acid receptor required to maintain GI integrity. In the small intestine, FXR maintains the expression of homeostasis pathways such as fibroblast growth factor 19 [113]. Fiorucci et al. [82] reported that NSAIDs, such as Indomethacin and Ketoprofen, reduced gastric H_2_S generation and CSE expression/activity in the gastric mucosa of male Wistar rats [82]. The reduction in H_2_S formation was seen to exacerbate mucosal damage; however, the treatment of rats with NaHS significantly alleviated the gastric injuries induced by these drugs. This finding demonstrates that H_2_S is an important component in maintaining GI integrity mediated by the KATP channel. Other researchers have shown that Diclofenac reduced serum H_2_S concentrations and the expression of CSE and CBS in the stomach of male Wistar rats, and that the novel H_2_S-releasing Diclofenac derivative ATB-337 reduced gastrointestinal-injury in animals [84]. This protective observation accounted for H_2_S production from this molecule, which inhibited the expression of TNF-α and the adherence of leukocyte to the vascular endothelium of mesenteric. Again, this finding points to an important role of H_2_S in the GI tract.

By far, the most widely studied drug known to impact mammalian H_2_S production is the COX-2 inhibitor, Aspirin (ASP). Yang et al. [90] reported that intragastrical administration of ASP to male Kunming mice significantly reduced H_2_S production in the gastric mucosa and caused gastric mucosal injury and lesion formation. Furthermore, in ASP-damaged tissues, the infiltration of inflammatory cells and resultant production of IL-6 and TNF-α, as well as oxidative markers, viz., myeloperoxidase (MPO) induction and GSH depletion, were reported. ASP significantly inhibited H_2_S generation and CSE expression in the gastric mucosa. Interestingly, the negative effects observed for ASP were mitigated using a pH-controlled H_2_S donor, JK-1, to replenish endogenous H_2_S levels. JK-1 reduced ASP-induced gastric lesion formation by lowering the levels of IL-6 and TNF-α production, by inhibiting oxidative stress and by inducing tissue GSH concentrations in the mouse gastric mucosa. Similarly, in mice administered with ASP, CBS and CSE mRNA levels were significantly reduced in gastric tissues by as much as 60–70%, and these reductions correlated with the severity of gastric injury [87]. Moreover, replenishment of H_2_S tissue levels using Na_2_S preserved GI integrity, prevented ASP mediated tissue damage, and was accompanied with the activity of GPBAR1, a receptor expressed in the GI that has a role in maintaining GI barrier integrity. In female albino Swiss mice, ASP significantly reduced H_2_S levels in the liver of animals compared to the control (1.52 to 1.15 nmol/g wet weight, control vs. ASP; *p* < 0.001) [85]. In male Wistar rats, ASP (125 mg/kg/ig) was reported to cause reductions in the CSE protein expression and H_2_S production of treated animals [88]. Interestingly, this study reported a significant elevation in CBS protein expression in gastric mucosal tissues. The upregulation of CBS can be explained as a compensatory mechanism in the GI that likely results from diminished H_2_S levels. Furthermore, pre-treatment with NaHS prevented damage induced by ASP by increasing the gastric bloodflow in treated animals [88].

In male Sprague-Dawley rats treated with ASP, a significant reduction in H_2_S concentration in gastric tissues was reported but no effects on plasma H_2_S concentrations were noted [86]. Researchers have indicated that as H_2_S-releasing derivatives of ASP, ACS14 protects against gastric damage induced by ASP. These protective effects occur by virtue of its H_2_S-releasing capacity. H_2_S production by this molecule was found to inhibit oxidative stress, viz., inhibition of gastric malondialdehyde (MDA) levels and to increase tissue GSH levels. In contrast, some studies have shown that ASP can increase H_2_S concentrations in the liver and brains of mice; however, it is unclear why this may be the case [83].

#### 1.2.2. Paracetamol

Paracetamol (*N*-acetyl-para-aminophenol; APAP) is a commonly used analgesic among the general population worldwide [114]. It is widely used among pediatric and adult populations for the treatment of pain and fever [115]. Paracetamol is a well-tolerated and remarkedly safe drug if used within the recommended dose, viz., 4 g/d [116]. Indeed, adults typically take from 0.5 to 1 g of oral Paracetamol every 4–6 h, up to a maximum of 4 g in a 24 h period [117]. Beyond this, the number of cases of liver damage caused by Paracetamol is steadily increasing every year worldwide [118]. Studies have shown that most of the toxic cases linked to Paracetamol, including acute liver failure, were due to chronic poisoning [119]. However, although physiological changes with the aging process can impact Paracetamol pharmacokinetics, no specific dose recommendations were advised for older people [120]. The elderly may be at greater risk of Paracetamol toxicity. Mitchell et al. [121] reported on the reductions in serum Paracetamol concentrations among old participants (70 years and above) compared to younger people (18–55 years) after 5 days of 3–4 g/d of oral Paracetamol consumption (*p* < 0.004). This may indicate reductions in Paracetamol clearance with aging [122].

Research on Paracetamol has spanned several decades since its introduction to clinical use in 1950 [123]. This molecule is known for its analgesic and antipyretic properties, which are similar to NSAIDs. There is little evidence of Paracetamol having any anti-inflammatory properties [124].

Recent evidence has emerged showing that APAP can impact H_2_S levels in mammals, although the data are quite sparse. In studies assessing the role of JNK/MAPK signaling in APAP-induced acute liver failure, Li et al. [93] reported that in male C57BL/6J mice, APAP, reduced the protein expression of both CBS and CSE in liver tissues. Moreover, pre-treatment with the H_2_S donor NaHS preserved tissue CSE and CBS expression levels in animals and reduced APAP toxicity. Molecular interrogation showed that H_2_S inhibited APAP-induced JNK activation in vivo. Moreover, in human hepatocyte cell lines (7702) treated with H_2_S, the activation of caspase 3, Bax, and Bcl-2 expressions were inhibited, and the phosphorylation status of JNK was reduced in hepatocytes. Combined, the protective effects of H_2_S were linked to the inhibition of apoptosis via suppression of the JNK/MAPK signaling pathway.

Similarly, in Wistar rats treated with APAP, CBS and glutathione synthase enzyme expression were reduced in the livers of treated animals, and this corresponded with diminished liver GSH levels and hepatocellular damage (*p* < 0.05) [80]. Whereas H_2_S levels were not measured in this study, it is interesting to speculate that the reduced capacity of CBS to generate H_2_S could have added to the damage caused by APAP treatment. It is now widely known that H_2_S is protective in models of APAP toxicity. Indeed, H_2_S can stimulate cysteine transport and increase hemeoxygenase (HO-1) expression and GSH concentration in tissues [125,126]. These effects prevent elevations in serum ALT, reduce the levels of hepatic MDA, and preserve GSH. Interestingly, co-treatment with *N*-acetylcysteine (NAC), a H_2_S-releasing molecule [127], significantly reduces MDA levels, increases tissue GSH levels and the expression of CBS in the livers of APAP treated animals. Furthermore, several studies have now shown that H_2_S supplementation in combination with APAP prevents toxicity in animals. Treatment with NaHS prevented APAP-induced kidney injury and preserved glomerular structures and function in Wistar albino rats administered APAP [128]. The protective effects were linked to the reduction in acute kidney injury (AKI) makers such as KIM-1 (Kidney Injury Molecule-1) and NGAL (neutrophil gelatinase-associated lipocalin). Other notable changes included reductions in the levels of TNF-α, TGFβ, and apoptosis in tissues. This work suggests that in this model, H_2_S had an anti-inflammatory effect in the kidney. Similarly, Morsy et al. [129] showed that administration of NaHS to Swiss mice treated with APAP had a significant reduction in the serum ALT levels and TNF-α expression in liver, in addition to reductions in hepatic MDA and nitric oxide (NO) [129]. This research shows that H_2_S had an anti-inflammatory effect in animals, leading to reductions in APAP-induced hepatoxicity. Other studies using CSE knockout animals have also pointed to roles of H_2_S in APAP-mediated toxicity in animals. The administration of NaHS was reported to suppress markers of liver damage in CSE KO mice, viz., ALT, AST, lactate dehydrogenase (LDH), and MDA in animals administered APAP. Researchers concluded that H_2_S reduced hepatotoxicity directly through the scavenging of N-acetyl-p-benzoquinoneimine (NAPQI) [130]. Other studies have shown that APAP reduces H_2_S levels in the brain of animals. Wiliński et al. [92] showed that in CBA-strain female mice, mice treated with 30 mg/kg/d or 100 mg/kg/d of APAP for five days had significantly reduced brain H_2_S concentration compared to a control group (1.47 ± 0.02 µg/g vs. 0.80 ± 0.02 µg/g vs. 1.05 ± 0.02 µg/g; *p* < 0.01: control vs. 30 mg vs. 100 mg) [92]. Collectively, of the available evidence, APAP appears to reduce the expression of H_2_S biosynthetic tissues in mammals, and the use of H_2_S donor molecules seems to prevent hepatotoxicity. Clearly, additional studies are warranted to further explore the molecular mechanisms of action of H_2_S in APAP-mediated toxicity.

#### 1.2.3. Anticancer Drugs

Few studies have explored the impact of chemotherapeutic drugs on H_2_S biosynthetic pathways in mammalian systems. What is currently known points to an interesting picture of H_2_S dysregulation that deserves further research. Cisplatin (*cis*-Diamminedichloroplatinum) is one of the most potent anticancer drugs for the treatment of solid cancers such as testicular, ovarian, head and neck, bladder, lung, cervical cancer, melanoma, and lymphoma, and is used as a first-line medicine in tumor treatment [131,132]. This molecule exerts its anticancer activity via several mechanisms, primarily by causing DNA lesions by interacting with purine bases [133]. In turn, the formation of Cisplatin DNA interactions drives the induction of signal transduction cascades linked to apoptosis in cells and tissues [134]. In patients, Cisplatin use is linked to the development of renal damage in 30% of patients [135]. It is only recently that studies have shown a direct link between Cisplatin-mediated toxicity and H_2_S. In male C57BL/6 mice exposed to Cisplatin, this drug promoted a decrease in expression for both CBS and CSE in the kidneys of treated animals (*p* < 0.01). Moreover, pre-treatment with GYY4137, a slow-release H_2_S donor molecule, aggravated renal injury induction through an elevation in the inflammatory response and the promotion of apoptosis [95]. In male Wistar rats, Cisplatin induced H_2_S formation in tissues and increased the expression of CSE in renal tissues of animals [94]. Moreover, the inhibition of CSE using DL-propargylglycine (PAG) prevented renal damage induced by Cisplatin. The reduction in renal damage correlated with diminished levels of apoptotic cells and the production of the proinflammatory molecule TNF-α levels in renal tissues. In contrast, Cao et al. [29] indicated that Cisplatin treatment in vitro significantly reduced the expression level of CSE in renal proximal tubular cells, leading to an impairment of H_2_S production in renal tissues, which likely contributed to renal toxicity. Moreover, NaHS treatment alleviated renal dysfunction. The molecular mechanism for these protective effects contributed to the reduction in ROS production.

#### 1.2.4. Statins

Statins are prescribed for the management of high cholesterol levels in individuals due to their effects at reducing low-density lipoprotein (LDL) cholesterol levels in the blood [136,137]. Statins function by inhibiting 3-hydroxy-3-methylglutaryl-coenzyme A (HMG-CoA) reductase, which enhances the rate-limiting step in the biosynthesis of cholesterol [138]. To date, approximately 145.8 million people use statins across 83 countries [139]. In the UK, around one in three people aged 45 years or over take statins [140]. Clear benefits have been reported for the use of these drugs to reduce the relative risk of cardiovascular events in the general population (reviewed by Sirtori [141]. However, several reported side effects are known, including muscle toxicity and liver enzyme effects [142].

The impacts of statins on H_2_S-generating systems are complex, with research showing both inhibitory as well as stimulatory impacts in animal and cell culture models. In murine Raw 264.7 macrophages treated with Fluvastatin or Atorvastatin, the mRNA and protein expression levels of CSE were increased in concentration- and time-dependent manners [98]. These changes correlated with parallel elevations in H_2_S in stimulated macrophages. Importantly, the inhibition of CSE with using dl-propargylglycine (PAG) or siRNA markedly reduced the H_2_S production in Fluvastatin-treated cells. Moreover, the PI3K inhibitor LY294002 and Akt inhibitor perifosine were able to reverse the increases of CSE mRNA and H_2_S production in Fluvastatin-stimulated macrophages. These observations confirm the role of P13 kinases and the AKT signaling pathway in H_2_S production. Other research has shown that in female CBA mice, Atorvastatin treatment (5 mg/kg/day) and (20 mg/kg/day) for five days decreased the level of H_2_S in the liver tissue of animals (3.45 ± 0.03 vs. 3.27 ± 0.02 vs. 3.31 ± 0.02; *p* < 0.01: control vs. 5 mg vs. 20 mg), but increased the levels of H_2_S in the kidney, brain, and heart tissues of animals [96]. This pattern indicates the complexity of H_2_S biosynthetic systems in different organs systems and their sensitivity to drugs. Similarly, Wójcicka et al. [97] showed that Pravastatin and Atorvastatin increased H_2_S production in the liver of animals by 51.7% and 70.7% in male Wistar rats, respectively [97]. Interestingly, the observed increase in H_2_S was associated with reductions in the mitochondrial oxidation of this gas. Furthermore, several novel H_2_S sensitive probes have been developed to explore the dynamics of H_2_S production in cells following statin treatment. For example, Zhang et al. [143] developed a near-infrared (NIR) fluorescence emission probe sensitive to H_2_S with high selectivity and sensitivity [143]. The developed probe, NIRDCM-H_2_S, was composed of a dicyanomethylene-4H-pyran (DCM) chromophore as the NIR fluorescence reporter and a pyridine-disulfide-propionate group as the responsive site toward H_2_S. This probe could be applied to the monitoring of endogenous production of H_2_S in raw264.7 macrophages in response to Fluvastatin treatment. Fluvastatin was found to promote the activity of CSE and the generation of H_2_S in murine macrophages. Other probes included an encapsulated semi-cyanine-BODIPY hybrid dye (BODInD-Cl) and its complementary energy donor (BODIPY1) into the hydrophobic interior of an amphiphilic copolymer (mPEG-DSPE). The developed radiometric fluorescent H_2_S nanoprobe was used in the trapping of endogenous H_2_S generation in raw264.7 macrophages upon stimulation with Fluvastatin. In parallel to previous reports, Fluvastatin induced CSE upregulation, leading to increased endogenous H_2_S generation in Fluvastatin-stimulated macrophages. These increases correlated with the activation of the Akt signaling pathway [144].

#### 1.2.5. Glucocorticoid

Glucocorticoids are a group of medications that has anti-inflammatory and immunosuppressive effects. This class of drug works via interactions with the glucocorticoid receptor in various cell types [145]. Glucocorticoids have been used widely since the 1950s to treat many diseases such as autoimmune disorders, asthma, inflammatory bowel diseases, and rheumatoid diseases. However, this medication group has adverse effects associated with long-term use, such as osteoporosis and muscle atrophy [146,147].

In an in vivo study, the expressions of CBS and CSE were downregulated following 8-day treatment with Dexamethasone, as well as the production of H_2_S in mesenteric carotid arteries in male Wistar rats (*p* < 0.05) compared to vehicle group. The observed reduction in H_2_S was accounted for the impairment of CBS and CSE expression, which led to an increase in the risk of hypertension [79]. This finding suggests that the inhibition of H_2_S production induced by Dexamethasone contributes to hypertension development in rats. Tai et al. [148] found that N-acetylcysteine (NAC), a reported H_2_S donor, could inhibited Dexamethasone-induced hypertension in adult male rats. The protective effect of NAC appeared to be linked to its capacity to increase plasma GSH, decrease oxidative stress, and increase protein levels of 3-MST in renal tissues of rats.

Similarly, evidence from several in vitro studies have shown Dexamethasone can suppress LPS-induced CSE expression and the production of H_2_S and NO in macrophage cells. Indeed, l-arginine increased, whereas N(G)-nitro-l-arginine methyl ester (l-NAME) decreased LPS-induced CSE expression and H_2_S production [99]. Moreover, the results suggest that H_2_S may exert anti-inflammatory effects by inhibiting NO production and that Dexamethasone can inhibit CSE expression and H_2_S production. Li et al. [28] showed that Dexamethasone administered either 1 h before or 1 h after LPS administration in rats inhibited the rise in plasma cytokine (interleukin [IL]-1β, tumor necrosis factor [TNF]-α), nitrate/nitrite (NO×), soluble intercellular adhesion molecule-1 (sICAM-1) concentrations, and lung/liver myeloperoxidase activity in animals, which is indicative of an anti-inflammatory effect [28]. However, it was also noted that Dexamethasone treatment reduced H_2_S concentrations in both plasma and liver tissues of male Sprague-Dawley rats. In human fetal liver cells and in isolated rat neutrophils, Dexamethasone also reduced the LPS-induced upregulation of CSE in cells. In chicken myoblasts treated with Dexamethasone, Dexamethasone was reported to inhibit protein synthesis, downregulate mTOR and p70S6K phosphorylation, and suppress the expression of the CSE protein in myoblasts. Moreover, L-cysteine and NaHS could abolish the inhibitory effects of Dexamethasone [100]. The abovementioned study indicates that H_2_S plays an important role in the skeletal muscle response to Dexamethasone by suppressing the protein synthesis induced by Dexamethasone. In murine calvaria-derived osteoblastic MC3T3-E1 cells, Dexamethasone downregulated CSE and CBS in osteoblastic cells. Interestingly, NaHS pre-treatment reduced Dexamethasone-induced apoptosis and LDH leakage. In this instance, H_2_S was reported to significantly activate AMPK signaling via the inhibition of ROS production and ATP depletion [101]. Other studies have reported on how Dexamethasone-induced bone loss is associated with decreased levels of serum H_2_S. Reductions in H_2_S were linked to losses in two key H_2_S-generating enzymes in the bone marrow, namely CBS and CSE. Interestingly, treatment with the H_2_S donor GYY4137 prevented a Dexamethasone-induced loss in bone formation. Mechanistically, GYY4137 promoted osteoblastogenesis by activating Wnt signaling through the increased production of the Wnt ligands. In comparison, blockage of the Wnt/β-catenin signaling pathway significantly alleviated the effect of H_2_S on osteoblasts. Collectively, findings from this study have inspired the development of novel H_2_S releasing steroidal therapeutics. Indeed, Corvino et al. [149] recently described the design, synthesis, and pharmacological assessment of novel glucocorticoids-H_2_S donor molecules. These compounds were based on two corticosteroids, namely Prednisone and Dexamethasone, that contained a H_2_S-donating moiety, consisting of 4-hydroxy-thiobenzamide (TBZ) and 5-(p-hydroxyphenyl)-1,2-dithione-3-thione (ADT-OH) residues. Glucocorticoids-H_2_S donors demonstrated prolonged chemical stability at both acidic and physiological pH levels, as well as the capacity to effectively inhibit mast cell degranulation. When assessed in vivo, H_2_S-releasing molecules could reduce the peribronchiolar density of collagen as well as the thickness of the smooth muscle actin better than prednisone.

#### 1.2.6. Phosphodiesterase (PDEs) Inhibitors

Phosphodiesterase (PDE) is a group of enzymes that breaks down the cyclic nucleotides of cAMP and cGMP. Therefore, PDE inhibitors increase intracellular cAMP and/or cGMP, causing muscle relaxation (vasodilatory effect) [103]. PDE inhibitors are a class of medications that have 11 types (PDE1-PDE11) prescribed for the management of conditions such as lower urinary tract symptoms (LUTS), chronic obstructive pulmonary disease (COPD), erectile dysfunction, and atopic dermatitis [102,150]. Headache and gastrointestinal problems such as nausea are commonly reported side effects [151].

Recently, an association between H_2_S levels and PDE inhibitor usage was reported. In an in vitro study, Fusco et al. [102] reported that Sildenafil, a PDE-5, relaxed the human bladder tissue in concentration- and time-dependent manners. However, pre-incubation with DL-propargylglycine (PAG) and aminooxyacetic acid (AOAA), CBS and CSE inhibitors, significantly reduced Sildenafil-induced relaxation (*p* < 0.001). Moreover, treatment with Sildenafil was seen to significantly increase H_2_S production in a concentration- and time-dependent manners in the human bladder dome. In contrast, CBS and CSE inhibition using PAG and AOAA was observed to significantly decrease Sildenafil-induced H_2_S production. This result suggests the involvement of H_2_S in Sildenafil-induced bladder muscle dilations. In pig and human bladder neck, Ribeiro et al. [103] observed that Rolipram, a PDE-4 type inhibitor, had concentration-dependent relaxation effects in tissues. However, the relaxation effect of Rolipram was reduced by blunting H_2_S production with the CSE inhibitor (PAG). This observation suggests the relaxation response is generated partly via H_2_S production in bladder tissues. Similarly, Agis-Torres et al. [104] reported that Rofumilast, a PDE-4, significantly elevated H_2_S production in pig bladder neck samples. When considered together, these findings reinforce a possible role for the H_2_S pathway in the action mechanism of PDE inhibitors in muscle relaxations to manage various diseases.

## 2. Conclusions

The impacts of common medications on cellular and tissue H_2_S formation are becoming more widely reported. Many of these animal and cell studies have indicated that several classes of drugs impact H_2_S homeostatic systems by decreasing the expression of enzymes needed for H_2_S biosynthesis such as CSE and CBS. Other studies have shown that some therapeutics reduce the mitochondrial oxidation of H_2_S in the mitochondria. The available evidence shows that two opposing systems are affected in mammalian systems, both critical in maintaining levels of H_2_S in vivo. Less widely known is whether the changes in H_2_S levels precipitate some of the known side effects of many commonly used drugs or if the replenishment of H_2_S using H_2_S donor molecules such as GYY4137 can mitigate potential side effects. In addition, there is a lack of information relating to whether changes in H_2_S production following drug treatment differ between organ and tissue systems or if compensatory mechanisms are induced to address the loss in function of individual H_2_S biosynthetic enzymes. Indeed, Atorvastatin elevates H_2_S formation in periaortic adipose tissue and liver of animals; however, it has little effect in aortic tissues. Importantly, as far as the authors are aware, there have been no intervention studies to study how drugs such as Paracetamol or NSAIDs impact the circulatory levels of H_2_S in humans. Clearly, further research is needed in this area to help clarify the significance of H_2_S dysregulation in tissues caused by common drugs.

## Figures and Tables

**Table 1:** Studies utilizing animal and/or cell-culture models to explore the impacts of common pharmacological drugs on H_2_S homeostasis.

Drug Used	Study Type	Consequence	Reference
NSAID: Indomethacin (10 mg/kg/day) and Ketoprofen (30 mg/kg/day)	Animal study: male Wistar rats	↓ Gastric H_2_S generation and CSE expression/activity in the gastric mucosa of rat, leading to exacerbate mucosal damage.	[82]
Aspirin (10 mg during five days)	Animal study: mice	↑ H_2_S in liver and brain of mice.	[83]
NSAID: Diclofenac (50 μmol/kg/day)	Animal study: male Wistar rats	↓ Serum H_2_S and ↓ expression of CSE and CBS in the stomach of male Wistar rats	[84]
NSAID: Aspirin (10 mg/kg/ip)	Animal study: female albino Swiss mice	↓ H_2_S levels in the liver of animals	[85]
NSAID: Aspirin (10–100 mg/kg); Indomethacin (10 mg/kg); Diclofenac (100 mg/kg); or Ketoprofen (30 mg/kg),	Animal study: mice	↓ CSE expression in the GI tract enhances susceptibility of FXR^−/−^ mice to damages caused by Aspirin and NSAIDs	[81]
Aspirin (200 mg/kg)	Animal study: male Sprague-Dawley rats	↓ H_2_S concentration in gastric tissues but had no effect on plasma H_2_S concentrations	[86]
Aspirin (50 mg/kg/day)	Animal study: mice	↓ Gastric expression of CBS and CSE mRNA by 60–70% leading to gastric injury.	[87]
Aspirin (125 mg/kg/ig)	Animal study: male Wistar rats	↓ CSE protein expression and H_2_S production, and ↑ CBS protein expression, in gastric mucosal tissues, causing gastric lesions.	[88]
NSAID: Naproxen (20 mg/kg/day)	Animal study: Wistar rats	↑ Gastric mucosal protein expression of CSE was observed when compared to controls, while no effect was noticed on CBS and 3-MST.	[89]
Aspirin (200 mg/kg/day)	Animal study: male Kunming mice	↓ H_2_S production in the gastric mucosa and caused gastric mucosal injury.↓ gastric GSH levels leading to dysregulate the endogenous redox status.	[90]
NSAID: Ketoprofen (10 mg/kg/day)	Animal study: rats	↓ H_2_S levels in the gastric and intestinal mucosa, leading to GI toxicity.	[91]
Paracetamol (30 mg/kg/d) or (100 mg/kg/d)	Animal study: CBA stain female mice	↓ brain H_2_S concentration compared to a control group.	[92]
Paracetamol (150 mg/kg)	Animal study: Wistar rats	↓ CBS and glutathione synthase enzyme expression in the liver of treated animals	[80]
Paracetamol (150 mg/kg/ip)	Animal study: male C57BL/6J mice	Animal study: ↓ Protein expression of both CBS and CSE In liver tissues.	[93]
Anticancer drug: Cisplatin (5 mg/kg/ip)	Animal study: male Wistar rats	↑ H_2_S formation and CSE expression in renal tissues of animals.	[94]
Anticancer drug: Cisplatin (20 mg/kg/ip)	Animal study: male C57BL/6 mice	↓ both CBS and CSE expression in the kidneys.	[95]
Anticancer drug: Cisplatin	In vitro: renal proximal tubular cells	↓ Expression level of CSE and ↓ H_2_S production in renal cortex tissues, which may contribute to renal toxicity.	[29]
Lipid lowering drug: Atorvastatin (5 mg/kg/day and 20 mg/kg/day)	Animal study: female CBA-strain mice	↓ H_2_S in the liver tissue (*p* < 0.01), but ↑ H_2_S levels in the kidney, brain and heart tissues of animals.	[96]
Lipid lowering drug: Pravastatin (40 mg/kg/day) and Atorvastatin (20 mg/kg/day)	Animal study: male Wistar rats	↑ H_2_S production in the liver of animals by 51.7% and 70.7%.	[97]
Lipid lowering drug: Fluvastatin (5 μM) or Atorvastatin (100 μM)	In vitro: murine raw 264.7 macrophages	↑ mRNA and protein expression levels of CSE in concentration and time dependent manners.↑ H_2_S production in raw 264.7 macrophages.	[98]
Glucocorticoid: Dexamethasone (1.5 mg/kg/day)	Animal study: male Wistar rats	↓ the expression of CBS, CSE and H_2_S production in mesenteries; leading to increase blood pressure.	[79]
Glucocorticoid: Dexamethasone (1–1000 nmol/L)	In vitro: macrophages cells	↓ mRNA and protein levels of CSE and H_2_S production in macrophages	[99]
Glucocorticoid: Dexamethasone(Animal study: 1 mg/kg, i.p.(In vitro: 1–10 µM)	Animal study: male Sprague-Dawley rats.In vitro: human foetal liver cells and rat neutrophils.	Animal study: ↓ H_2_S concentration in both plasma and tissues.In vitro: ↓expression of CSE in both human foetal liver cells and in rat neutrophils.	[28]
Glucocorticoid: Dexamethasone (10 μM)	In vitro: chicken myoblasts	↓ expression of the CSE protein in myoblasts.↓mTOR and p70S6K phosphorylation.↓ protein synthesis.	[100]
Glucocorticoid: Dexamethasone (1 μM)	In vitro: murine calvaria-derived osteoblastic MC3T3-E1 cell line	↓ expression of both CBS and CSE in osteoblastic.	[101]
Phosphodiesterase-5 inhibitors: Sildenafil (1, 3, 10, and 30 μM)	In vitro: human tissue; bladder	↑ H_2_S production in concentration and time dependent manners in human bladder dome.	[102]
Phosphodiesterase-4 inhibitors: Rolipram (0.1 nM–10 μM)	Pig and human bladder neck	- CSE inhibitor, DL-propargylglycine (PPG, 1 mM), ↓ the Rolipram relaxation.	[103]
Phosphodiesterase-4 inhibitors: Rolipram: Rofumilast (0.1, 1 and 10 µM)	Pig bladder neck	↑ H_2_S production in pig bladder neck samples	[104]

## Supplementary Materials

The following supporting information can be downloaded at: https://www.mdpi.com/article/10.3390/antiox12040908/s1, Table S1: Studies utilizing animal and/or cell-culture models to explore the impacts of common pharmacological drugs on H_2_S homeostasis.
