# Supplementary material for: The Impact of Drugs on Hydrogen Sulfide Homeostasis in Mammals
Source: Antioxidants (Basel). 2023 Apr 11;12(4):908. doi: 10.3390/antiox12040908 (PMC10135325; doi:10.3390/antiox12040908)
Supplement: Supplementary file 1 [file antioxidants-12-00908-s001.zip › antioxidants-2208686-supplementary.pdf]

Table 1. Studies utilizing animal and/or cell-culture models to explore the impacts of common pharmacological drugs on H_2S homeostasis.

Drug used	Study type	Consequence	Reference
NSAID: Indomethacin (10mg/kg/day) and Ketoprofen (30mg/kg/day)	Animal study: male Wistar rats	↓ Gastric H_2S generation and CSE expression/activity in the gastric mucosa of rat, leading to exacerbate mucosal damage.	[125]
Aspirin (10mg during five days)	Animal study: mice	↑ H_2S in liver and brain of mice.	[126]
NSAID: Diclofenac (50μmol/kg/day)	Animal study: male Wistar rats	↓ Serum H_2S and ↓ expression of CSE and CBS in the stomach of male Wistar rats	[127]
NSAID: Aspirin (10mg/kg/ip)	Animal study: female albino Swiss mice	↓ H_2S levels in the liver of animals	[128]
NSAID: Aspirin (10–100mg/kg); Indomethacin (10mg/kg); Diclofenac (100mg/kg); or Ketoprofen (30mg/kg),	Animal study: mice	↓ CSE expression in the GI tract enhances susceptibility of $FXR^{-/-}$ mice to damages caused by Aspirin and NSAIDs	[124]
Aspirin (200mg/kg)	Animal study: male Sprague-Dawley rats	↓ H_2S concentration in gastric tissues but had no effect on plasma H_2S concentrations	[129]
Aspirin (50mg/kg/day)	Animal study: mice	↓ Gastric expression of CBS and CSE mRNA by 60–70%, leading to gastric injury.	[130]
Aspirin 125mg/kg/ig	Animal study: Male Wistar rats	↓ CSE protein expression and H_2S production, and ↑ CBS protein expression in gastric mucosal tissues, causing gastric lesions.	[131]
NSAID: Naproxen (20mg/kg/day)	Animal study: Wistar rats	↑ Gastric mucosal protein expression of CSE was observed when compared to controls, while no effect was noticed on CBS and 3-MST.	[132]

Drug used	Study type	Consequence	Reference
Aspirin (200mg/kg/day)	Animal study: male Kunming mice	↓ H_2S production in the gastric mucosa and caused gastric mucosal injury.<br>↓ gastric GSH levels leading to dysregulate the endogenous redox status.	[133]
NSAID: Ketoprofen (10mg/kg/day)	Animal study: rats	↓ H_2S levels in the gastric and intestinal mucosa, leading to GI toxicity.	[134]
Paracetamol (30mg/kg/d) or (100mg/kg/d)	Animal study: CBA-strain female mice	↓ brain H_2S concentration compared to a control group.	[135]
Paracetamol (150mg/kg)	Animal study: Wistar rats	↓ CBS and glutathione synthase enzyme expression in the liver of treated animals	[123]
Paracetamol (150mg/kg/ip)	Animal study: male C57BL/6J mice	Animal study: ↓ Protein expression of both CBS and CSE In liver tissues.	[136]
Anticancer drug: Cisplatin (5mg/kg/ip)	Animal study: male Wistar rats	↑ H_2S formation and CSE expression in renal tissues of animals.	[137]
Anticancer drug: Cisplatin (20mg/kg/ip)	Animal study: male C57BL/6 mice	↓ both CBS and CSE expression in the kidneys.	[138]
Anticancer drug: Cisplatin	In vivo and<br>In vitro	↓ Expression level of CSE and ↓ H_2S production in renal cortex tissues, which may contribute to renal toxicity.	[58]
Lipid-lowering drug: Atorvastatin 5mg/kg/day and 20mg/kg/day	Animal study: female CBA-strain mice	↓ H_2S in the liver tissue ($p<0.01$), but ↑ H_2S levels in the kidney, brain, and heart tissues of animals.	[139]
Lipid-lowering drug: Pravastatin (40mg/kg/day) and Atorvastatin (20mg/kg/day)	Animal study: male Wistar rats	↑ H_2S production in the liver of animals by 51.7% and 70.7%.	[140]

Drug used	Study type	Consequence	Reference
Lipid lowering drug: Fluvastatin (5µM) or Atorvastatin (100µM)	*In vitro*: murine raw 264.7 macrophages	↑ mRNA and protein expression levels of CSE in concentration and time dependent manners.<br>↑ H_2S production in raw 264.7 macrophages.	[141]
Glucocorticoid: Dexamethasone (1.5mg/kg/day)	Animal study: male Wistar rats	↓ the expression of CBS, CSE and H_2S production in mesenteries; leading to increase blood pressure.	[122]
Glucocorticoid: Dexamethasone (1–1,000 nmol/l)	*In vitro*: macrophages cells	↓ mRNA and protein levels of CSE and H_2S production in macrophages	[142]
Glucocorticoid: Dexamethasone (Animal study: 1mg/kg, i.p. (*In vitro*: 1-10µM)	Animal study: Male Sprague-Dawley rats.<br>*In vitro*: human foetal liver cells and rat neutrophils.	Animal study: ↓ H_2S concentration in both plasma and tissues.<br>*In vitro*: ↓expression of CSE in both human foetal liver cells and in rat neutrophils.	[57]
Glucocorticoid: Dexamethasone (10µM)	*In vitro*: Chicken myoblasts	↓ expression of the CSE protein in myoblasts.<br>↓mTOR and p70S6K phosphorylation.<br>↓ protein synthesis.	[143]
Glucocorticoid: Dexamethasone (1µM)	*In vitro*: Murine calvaria-derived osteoblastic MC3T3-E1 cell line	↓ expression of both CBS and CSE in osteoblastic.	[144]
